# Supplementary material for: Analysis of PPARγ Signaling Activity in Psoriasis
Source: Int J Mol Sci. 2021 Aug 10;22(16):8603. doi: 10.3390/ijms22168603 (PMC8395241; doi:10.3390/ijms22168603)
Supplement: Supplementary file 1 [file ijms-22-08603-s001.zip › Supplemental materials_Analysis of PPARg signaling activity in psoriasis/Pathway models/Models images and html files/PPARG inhibits phenotypes assotiated with psoriasis/244071.html]

liver fibrosis


# Disease liver fibrosis

|  |  |
| --- | --- |
| URN | urn:agi-meshdis:Fibrosis%2c%20Liver |
| Total Entities | 2 |
| Connectivity | 3827 |
| Name | liver fibrosis |

---

|  |  |
| --- | --- |
| ChildConcepts | Thompson-Baraitser syndrome |
|  | experimental liver fibrosis |

---

|  |  |
| --- | --- |
| Pathway | PPARG inhibits phenotypes assotiated with psoriasis |
|  | Neighbors of liver fibrosis |

---

|  |  |
| --- | --- |
| MedScan ID | 9012637 |

---

|  |  |
| --- | --- |
| Alias | biliary fibrosis |
|  | intrahepatic fibroses |
|  | peri-portal fibrosis |
|  | intra-hepatic fibroses |
|  | Liver Fibroses |
|  | fibrous hepatic disease |
|  | Fibrosis, Liver |
|  | fibrotic biliary |
|  | periportal fibroses |
|  | liver periportal fibrosis |
|  | Fibroses, Liver |
|  | fibrotic liver |
|  | Liver Fibrosis |
|  | intrahepatic fibrosis |
|  | periportal fibrosis |
|  | intra-hepatic fibrosis |
|  | hepatic fibroses |
|  | fibrotic livers |
|  | fibrotic hepatic |
|  | hepatic fibrosis |
|  | cirrhotic liver |
|  | cirrhotic livers |

---

|  |  |
| --- | --- |
| MeSH ID | M0012637 |

---

|  |  |
| --- | --- |
| MeSH Heading | Fibrosis, Liver |

---
